# Synthesis of thermoelectric magnesium-silicide pastes for 3D printing, electrospinning and low-pressure spray

**DOI:** 10.1007/s40243-019-0159-7

**Published:** 2019-10-09

**Authors:** A. C. Marques, Davide Miglietta, G. Gaspar, A. C. Baptista, A. Gaspar, P. Perdigão, I. Soares, C. Bianchi, D. Sousa, B. M. Morais Faustino, V. S. Amaral, T. Santos, A. P. Gonçalves, R. C. da Silva, Fabrizio Giorgis, I. Ferreira

**Affiliations:** 1 CENIMAT/I3N, Departamento de Ciência dos Materiais, Faculdade de Ciências e Tecnologia, Universidade Nova de Lisboa, 2829-516 Caparica, Portugal; 2 Dipartimento di Scienza Applicata e Tecnologia, Politecnico di Torino, Corso Duca degli Abruzzi 24, 10129 Turin, Italy; 3 I3N/Departamento de Física e CICECO, Instituto de Materiais de Aveiro, Universidade de Aveiro, 3810-193 Aveiro, Portugal; 4 Departamento de Física e CICECO, Instituto de Materiais de Aveiro, Universidade de Aveiro, 3810-193 Aveiro, Portugal; 5 C2TN, Instituto Superior Técnico, Universidade de Lisboa, Campus Tecnológico e Nuclear, Estrada Nacional 10, 2695-066 Bobadela, LRS Portugal; 6 IPFN-IST/UL, Instituto de Plasmas e Fusão Nuclear, Instituto Superior Técnico, Universidade de Lisboa, Estrada Nacional 10, 2695-066 Bobadela, Portugal

**Keywords:** Mg_2_Si-based thermoelectric materials, Mechanical alloying, 3D printing, Electrospinning, Spray

## Abstract

In this work, eco-friendly magnesium-silicide (Mg_2_Si) semiconducting (*n*-type) thermoelectric pastes for building components concerning energy-harvesting devices through 3D printing, spray and electrospinning were synthetized and tested for the first time. The Mg_2_Si fine powders were obtained through the combination of ball milling and thermal annealing under Ar atmosphere. While the latter process was crucial for obtaining the desired Mg_2_Si phase, the ball milling was indispensable for homogenizing and reducing the grain size of the powders. The synthetized Mg_2_Si powders exhibited a large Seebeck coefficient of ~ 487 µV/K and were blended with a polymeric solution in different mass ratios to adjust the paste viscosity to the different requirements of 3D printing, electrospinning and low-pressure spray. The materials produced in every single stage of the paste synthesis were characterized by a variety of techniques that unequivocally prove their viability for producing thermoelectric parts and components. These can certainly trigger further research and development in green thermoelectric generators (TEGs) capable of adopting any form or shape with enhanced thermoelectric properties. These green TEGs are meant to compete with common toxic materials such as Bi_2_Te_3_, PbTe and CoSb that have Seebeck coefficients in the range of ~ 290–700 μV/K, similar to that of the produced Mg_2_Si powders and lower than that of 3D printed bulk Mg_2_Si pieces, measured to be ~ 4866 μV/K. Also, their measured thermal conductivities proved to be significantly lower (~ 0.2 W/mK) than that reported for Mg_2_Si (≥ 4 W/mK). However, it is herein demonstrated that such thermoelectric properties are not stable over time. Pressureless sintering proved to be indispensable, but difficultly achievable by long thermal annealing (even above 32 h) in inert atmosphere at 400 °C, at least for bulk Mg_2_Si pieces constituted by a mean grain size of 2–3 μm. Hence, for overcoming this sintering challenge and become the silicide’s extrusion viable in the production of bulk thermoelectric parts, alternative pressureless sintering methods will have to be further explored.

## Introduction

The increasing energy demand worldwide has been driving the search for new, clean, renewable and sustainable energy sources. Solar, wind and hydropower energy sources are expected to fulfill future energy needs and replace energy sources based on fossil fuels. However, currently, these still assure about 90% of the world’s electricity generation with low operating efficiency (30–40%) and large annual waste of heat to the environment (15 TW) [1]. Such large amount of wasted heat can be directly converted into electricity by solid-state generators based on the thermoelectric (TE) materials, using the Seebeck effect. Nowadays, thermoelectric generators (TEG) are already powering a number of devices in a very broad field of applications, ranging from medical, military and space applications, infrared sensors, computer chips, battery charging, waste heat recovery (e.g. from car exhausts) to rural home electrification [2–4]. Although TEGs have many advantages such as of compactness, low complexity, high reliability and silent operation (no moving parts), low maintenance cost and environmental compatibility of operation, they are not massively used due to their low TE conversion efficiency (< 10%). In fact, TEGs are actually used only in niche markets where the reliability is more important than performance and cost is not a main consideration [5]. Some of the issues with current TEGs hindering their proliferation are the lack of stability at extremes temperatures, along with problems of environmental friendliness, availability, and high costs of the base materials and the synthesis. Therefore, materials such as Mg_2_Si have recently attracted much attention: these alloys have been demonstrated as good TEG candidate base materials as their synthesis has become easier and achievable by a variety of methods, their constituent elements are non-toxic (contrarily to direct competitors such as PbTe and CoSb_3_), abundant and light weight. The base silicide thermoelectric properties can be enhanced and tuned through doping, increasing the conversion efficiency in many applications (e.g. industrial furnaces, automobile exhausts, and incinerators in the mid-temperature range 230–730°C). For instance, Mg_2_Si doped with Sb, Al and Bi has been used for the low and high temperature ends, respectively [6], while double doping allows higher figures of merit (ZT), currently in the range of 0.8–1.1 [7, 8], with new developments promising ZT values higher than 1.6 [9]. However, an important issue affecting Mg_2_Si-based TEGs is the lack of shape control of the traditional synthesis methods, mostly relaying on ingots formation. This makes it difficult or even impossible to properly adapt to curved heat sources, inevitably introducing higher thermal/heat transfer impedances, leading to considerable heat losses and lower energy conversion efficiencies of the devices. A new approach is herein devised to overcome the challenge of shape: it consists in the production of Mg_2_Si powders through a simple and cost-effective process (relying on the combination of ball milling with thermal annealing), for subsequent formulation of thermoelectric pastes suitable for 3D printing, electrospinning and spray technologies. The major problems with the Mg_2_Si powder synthesis and paste formulation are related to the high reactivity of Si and Mg powders with oxygen, demanding the use of an inert atmosphere, e.g. a glove box filled with Ar, and limits the selection of solvents and polymers to oxygen-free compounds. One should note that the need for developing thermoelectric parts with any form or shape is a very actual topic that has been differently addressed in other research works, for instance, through the development of Bi_2_Te_3_-based inorganic paints with Sb_2_Te_3_ as a sintering aid [10]. The Mg_2_Si pastes herein proposed can be a competitive alternative applicable in a broad range of TEG-based applications, e.g. from the automobile to the textile sectors, here in the form of woven fabrics of functional fibers.

## Materials and methods

Magnesium and silicon powders of less than 44 μm nominal grain size (mesh 325)—from Alfa Aesar with 99.8% and 99.5% purity, respectively—were loaded in a 2:1 mass ratio into a 50 mL agate bowl along with hexane and three 20 mm diameter agate balls to be mechanically alloyed in a high energy planetary ball mill (Retsch PM100). Hexane was added to prevent agglomeration of Mg powder on the walls and milling balls. The fluid and balls-to-powder mass ratios were 2:1 and 10:1, respectively. To reduce and homogenize the powder grain size, milling times of 2 h, 5 h and 10 h were tested. The powders and hexane fluid were weighted and transferred to the mill bowl inside a glove box filled with Ar gas. As the agate bowl is sealed inside the glove box with an o-ring fitting lid secured by a custom-made clamp, Ar will also be the atmosphere inside the bowl during the mill, avoiding oxidation of the reactants. The rotational speed was 400 rpm with 5 min pauses every 30 min, in all cases; after milling, the resulting powder was collected in the glove box and directly loaded in an alumina crucible for Ar thermal annealing at a flow rate of ~ 0.35 L/min. The holding temperature was set between 350 and 590°C, depending on the powders grain size. The annealing temperatures for the unmilled (590 °C), and the 2 h, 5 h (410 °C) and 10 h (350 °C) milled powders were defined from the differential scanning calorimetry (DSC) heat flow curves, simultaneously performed with thermogravimetric measurements (TGA). Both measurements were simultaneously performed in the thermal analyser STA 449 F3 Jupiter under different atmospheres (air and N_2_) from room temperature up to 1000 °C, at a rate of 20 K/min.

For all samples, the annealing temperature profiles consisted of a heating ramp of ~ 15 °C/min to the desired holding temperature, and a holding time of ~ 75 min, after which the temperature was ramped down to ~ 160 °C. Then, the furnace was turned off and the powders left to cool to room temperature.

The synthetized Mg_2_Si powders were mixed with polystyrene (PS)—from Sigma Aldrich, Mw ~ 350,000—in xylene solutions for obtaining *n*-type thermoelectric paste formulations—one per application method: 3D printing, spraying and electrospinning. For 3D printing, the Mg_2_Si powders were blended with a solution of 20% wt of polystyrene in xylene in the mass proportions of 43/57 (formulation 1) and 40/60 (formulation 2). These formulations were extruded in a home-adapted 3D printer equipped with a hot plate set to 50 °C to favor the fast evaporation of xylene. Fibers of Mg_2_Si were produced by low pressure N_2_-spray gun (Wuto) using a diluted version of formulation 2 and by electrospinning using a blend of Mg_2_Si powders with a 35% wt of PS solution in a mass proportion 7:93 (formulation 3). This was loaded into a syringe (B. Braun) connected to a blunt metallic needle with an internal diameter of 1.19 mm (18G from ITEC, Iberiana Technical). A syringe pump (NewEra SyringePump.com) was used to eject the solution at a controllable speed (0.2 mL/h) through the needle while a high voltage of 20 kV was applied (Glassman high voltage–power supply). A grounded Al static plate was placed at 15 cm from the needle to collect the fibers. A fourth paste was formulated with polyvinylidene difluoride (PVDF) solution in dimethylformamide (DMF) and then tested to produce bulk Mg_2_Si parts. The PVDF was heated together with DMF at 70 °C until PVDF is completely dissolved.

The temperatures at which the PS polymer can be burned out from the printed pieces were determined by DST/TGA to be ~ 460–470 °C (depending on the PS concentration). A holding time of ~ 90 min preceded by a heating ramp of 2–5 °C/min revealed to be enough for that end.

The morphology and composition of the milled powders, before and after annealing, were studied using a FEG-SEM Jeol JSM7001F and a Vega 3 TESCAN scanning electron microscopes (SEM), both equipped with an energy dispersive X-ray spectrometer (EDS). The crystalline phases were identified by X-ray diffraction (XRD) using a Panalytical X-PERT Powder diffraction unit, through Cu K_α_ radiation (*λ* = 0.1540598 nm). Confocal Raman spectrophotometer (Witec Alpha 300 RAS) using a laser with a wavelength of 532 nm and 4.11 mW of power was used to confirm the existence of the Mg_2_Si phase on the synthetized powders and also on both the printed pieces and fibers. The surface area, the pore volume and the average pore size were measured using Gemini V-2380 surface area analyser from Micromeritics and Gemini v2.0 software. The specific surface area (Brunauer-Emmett-Teller, BET, method) was determined from nitrogen adsorption isotherms determination for samples immersed in a liquid nitrogen bath. Barrett-Joyner-Halenda (BJH) method was used to calculate the pore size distribution in the samples. Prior to these measurements, the water vapor and adsorbed gas were removed by purging the samples in nitrogen flow for about 10 h. Over this period, the heat treatment of samples A and B was held at 120 °C, while for samples C at 300 °C.

The thermal conductivity was measured at room temperature (300 K) using the Gustafsson Probe method (Hot Disk) with the Thermal Constant Analyser TPS 2500 S. This method is based on the Transient Plane Source (TPS) technique and uses an electrically conductive double spiral flat sensor that is protected by a kapton 70 µm thick film, acting both as pulsed heat source and temperature sensor. The TPS was assembled between two similar 3D printed 10 mm diameter disks. The measured thermal conductivity is a result of 14 consecutive and equal measurements. All measurement parameters were double checked and the results were consistent, since the residuals of temperature data fitting as a function of time present a random scatter dispersion within a few 1.5 mK. This is also indicative of a good contact between the sensor and the twin samples, a stable temperature in the samples and that the heat pulse did not reach the sample boundary.

**Fig. 1:**
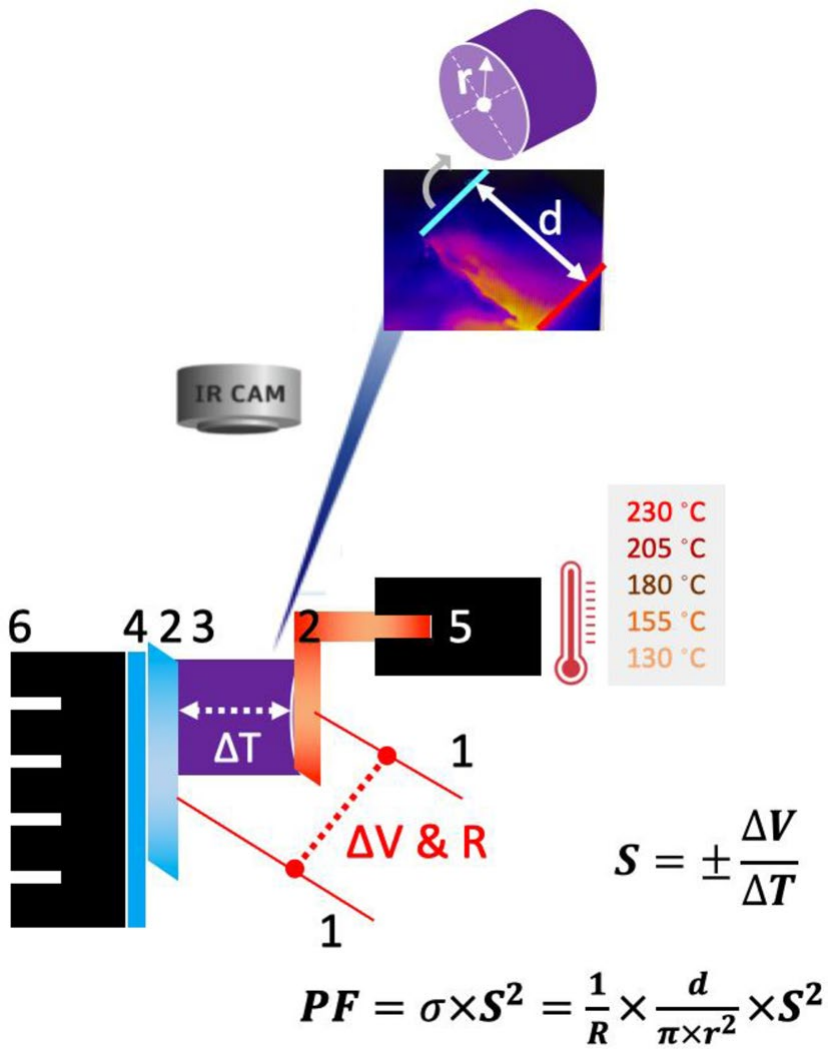
A schematic of the cross-sectional view of the home-made apparatus for the Seebeck measurements of bulk thermoelectrics. 1, Nano-voltmeter probes to measure the thermo-voltage; 2, conductive tapes attached to the C-paste-based electrodes on the cylinder faces; 3, Mg_2_Si thermoelectric cylinder; 4, cold peltier; 5, variable hot source; 6, heat sink

Electric and thermoelectric characterizations were performed with a home-made setup illustrated in Fig. 1. A temperature difference (ΔT) is imposed across the piece thickness using a heat source of variable temperature (from 130 to 230 °C in steps of 25 °C) and one TEC1-12707 Peltier module connected to an independent power source—meant to work as the cold source. The Seebeck coefficient was determined by imposing a ∆T and monitoring it with a FLIRA310 thermal camera, while the thermoelectric voltage (ΔV) was measured using an Agilent 34420A nano-voltmeter, using C-paste electrodes with conductive tapes on top and connecting to the cold/hot sources. The electrode area was ~ 70 mm^2^ and was separated by ~ 9 mm^2^. Seebeck coefficient was obtained from the slope of the plot ∆*V* versus ∆*T* as shown in Fig. 1, subsequently enabling the calculus of the power factor. A variable load resistance connected to the TE elements and Δ*V* measurements across terminals, from short-circuit to open-circuit conditions enable power output determination (*P*_out_ = *I*_out_ × *V*_out_).

## Results and discussion

### Mg_2_Si powder synthesis

**Fig. 2:**
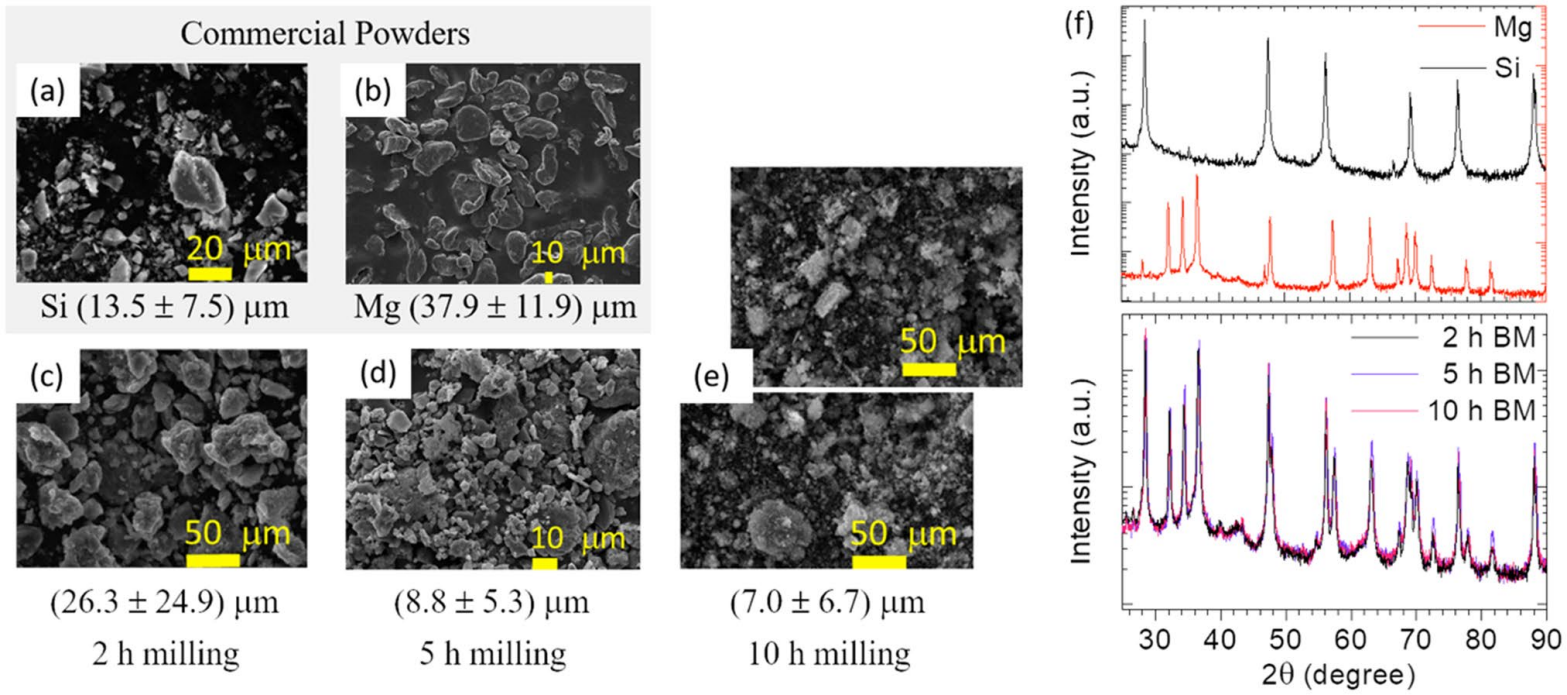
SEM micrographs of Si and Mg commercial powders before (**a**–**b**) and after milling for 2 h (**c**), 5 h (**d**) and 10 h (**e**). The average grain size is indicated together with the standard deviation. The XRD diffratograms of the starting Si and Mg powders (top) and the milled powders (bottom) are shown in (**f**)

**Fig. 3:**
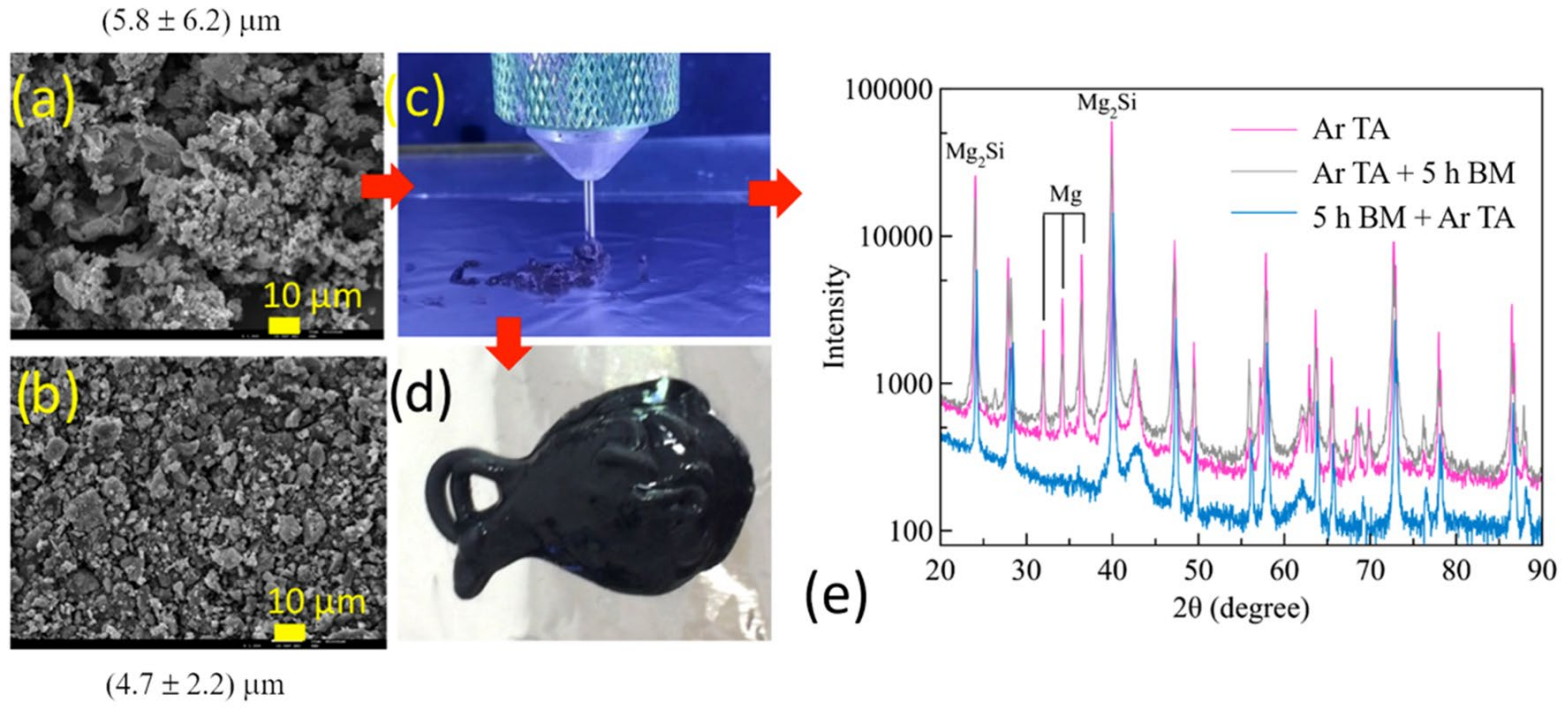
In the left half: SEM micrographs of Mg_2_Si powders obtained directly by thermal annealing only (**a**) and after the sequence thermal annealing and 5 h milling (**b**); photos of extruded Mg_2_Si paste made with powders formed through thermal annealing only (**c**) and close-up (**d**), respectively. In the right half: XRD diffratograms of the Mg_2_Si powders (**e**) obtained directly by thermal annealing (pink line) and by thermal annealing and subsequent 5 h milling (gray line). For comparison, the XRD measurements obtained from powders milled for 5 h and annealed is also included (blue line)

The SEM images shown in Fig. 2 illustrate the influence of ball milling in the particle size of Mg- and Si- powders. The purchased powders shown in figure (a) and (b) consist of irregularly shaped grains with a somewhat heterogeneous size distributions, with mean values and standard deviations of, respectively, ~ 13.5 μm and 7.5 μm for Si, and ~ 37.9 μm and 11.9 μm for Mg, in compliance with the stated 325 mesh specifications—implying a particle size distribution with upper limit of ~ 44 μm. After milling the Mg- and Si- powders in a 2:1 mass ratio for 2 h (Fig. 2c), 5 h (Fig. 2d) and 10 h (Fig. 2e), both shape and grain size distribution become more homogeneous, while the grain size was progressively reduced to ~ 26.3 ± 24.9 μm, ~ 8.8 ± 5.3 μm and ~ 7 ± 6.7 μm, respectively. The milling of the Mg- and Si- powder mixture was performed not only for reducing and homogenizing the grain size, aiming at enhancing the extrusion of pastes by 3D printing, spray and electrospinning, but also for verifying if any amount of the Mg_2_Si phase had formed as a result of the relatively short duration of the ball milling. Usually, much longer durations are required [11, 12]. XRD measurements on pristine Mg and Si powders, and mixtures of both, followed by 2 h, 5 h and 10 h milling are shown in Fig. 2. In all diffratograms, only the diffraction lines arising from Mg and Si are seen, no significant additional phases were detected (MgO fraction was below to 3%). Regardless the milling time, the XRD diffratograms from the milled powders can be described as a linear combination of XRD diffratograms of the pure Mg and Si commercial powders, with no trace of Mg_2_Si whatsoever. This observation, together with that presented in Fig. 2, clearly demonstrates that milling up to 10 h, does not lead to alloying; it merely decreases the grain sizes and homogenizes the size distributions. On the contrary, Fig. 3 XRD diffratograms clearly show that thermal annealing is of paramount importance for the formation of the Mg_2_Si phase. This can be easily obtained through thermal annealing (TA) only of the Mg- and Si- powders in a 2:1 mass ratio, even without ball milling (BM). The drawback is that Mg_2_Si powders produced in this way lead to a small amount of unreacted Mg and to grains agglomerations which, as illustrated in Fig. 3, made it impossible to 3D print the pastes formulation with such powders on the available printer, currently operating with needles of inner diameter of 0.61 mm and 1.19 mm. For mitigating this issue, the Mg_2_Si powders produced by directly annealing the Si–Mg powder mix were subsequently milled for 5 h to reduce and homogenize the particles, making them suitable for manufacturing 3D printing pastes. The XRD diffratogram of Mg_2_Si powders produced in the reverse sequence (i.e. 5 h BM → Ar TA) is also included in Fig. 3 for comparison. This does not exhibit the peaks corresponding to a fraction of unreacted Mg and moreover, quantification through the Rietveld refinement method yields an Mg_2_Si fraction of ~ 71.3% for this sequence. As shown in Table 1, similar fractions of Mg_2_Si were also formed through thermal annealing only (~ 76.3%) or in the sequence Ar TA → 5 h BM (~ 78.3%). Additional phases are due to unreacted Mg fast oxidization, leading to the MgO phase as well as unreacted Si, that can also react with O_2_ and lead to SiO_2_. The presence of some MgO along with the Mg_2_Si is not entirely surprising, since the insertion of the powder carrying crucibles in the quartz tubes used for annealing was always done in open air.

**Table 1:** XRD phases quantification of powders processed under different conditions

Powder synthesis sequence:	Qty. (%)				
	Mg_2_Si	MgO	Si	Mg	SiO_2_
Ar TA	76.3	8.3	1.4	14.1	–
5 h BM + Ar TA	71.3	11.1	7.0	–	10.6
Ar TA + 5 h BM	78.3	6.8	4.8	10.1	–

**Fig. 4:**
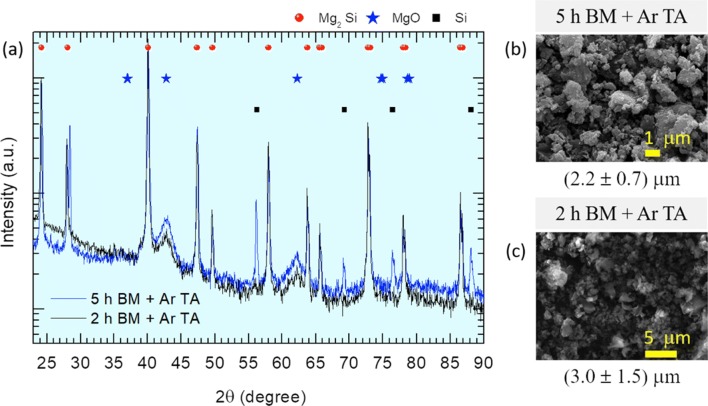
XRD diffratograms (**a**) of Si–Mg powders ball milled (BM) for 2 h (black line) and 5 h (blue line) and next thermally annealed in Ar. The SEM micrographs after the synthesis sequences are also shown in **b** and **c**. The average grain size taken from each micrograph is indicated together with the standard deviation

The sequence BM→ Ar TA was also performed for a shorter milling duration of 2 h as shown in Fig. 4a, the XRD diffratograms are compared with that of Mg–Si powders processed in the sequence 5 h BM → Ar TA. Both diffratograms are very similar which suggests that milling duration only impacts, as expected, in the final grain sizes. However, thermal annealing further reduced down the grain sizes of Mg–Si powders produced through 2 h BM → Ar TA to 3 μm, and 5 h BM→Ar TA to 2 μm, respectively. This is shown in the SEM micrographs of Fig. 4b, c and may be attributed to the release of high residual stresses which may have led to polygonization and hence to the formation of new small grains with more homogeneous microstructure.

### Mg_2_Si paste synthesis and application

**Fig. 5:**
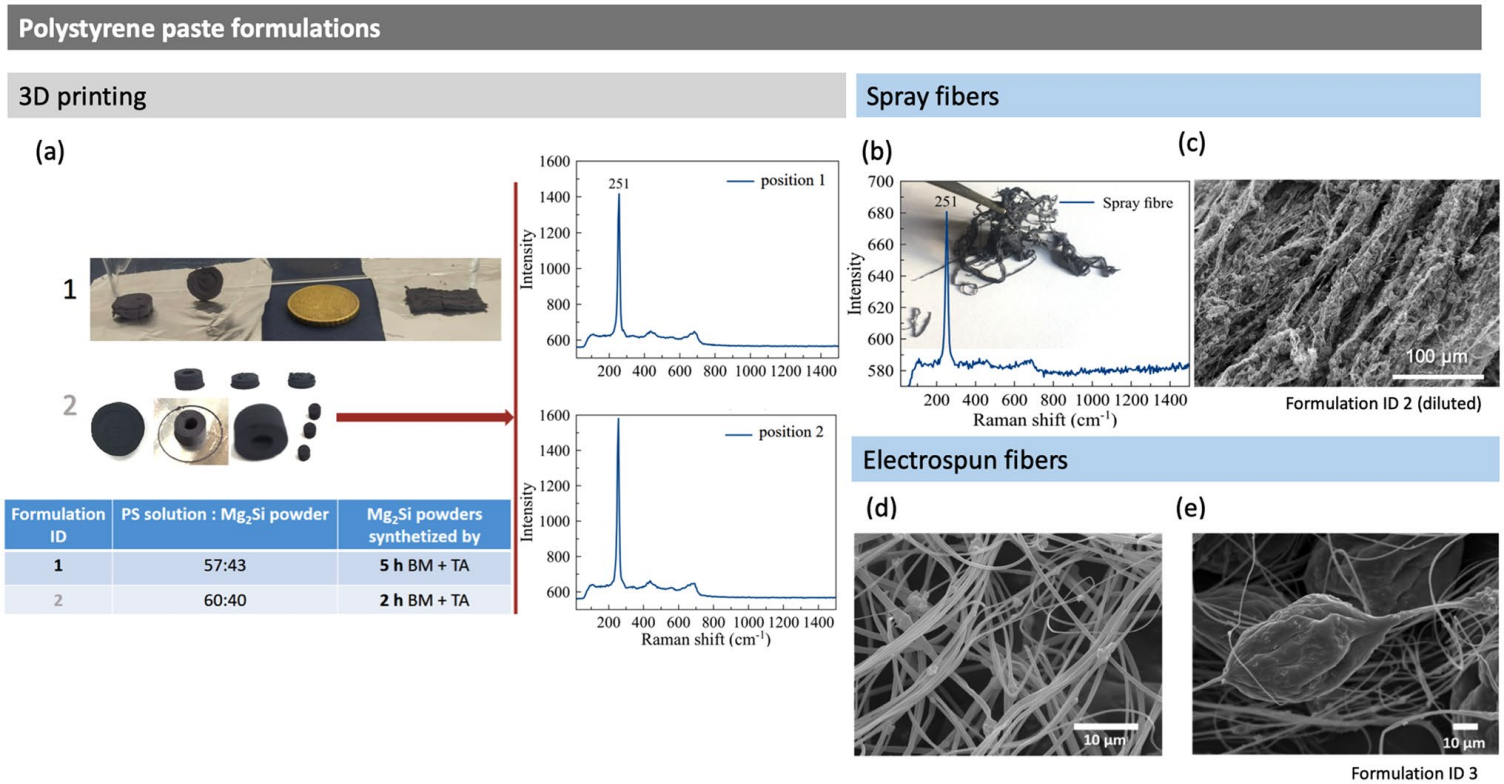
**a** Pieces printed with Mg_2_Si pastes following formulations 1 and 2 and SEM images of sprayed (**b**, **c**) and electrospun PS/Mg_2_Si fibers (**d**, **e**) produced with paste formulations 2 and 3, respectively. 3D-printing formulations are specified in Table 2

The high reactivity of Si and Mg powders with oxygen demands the use of solvents and polymers free of oxygen. Polystyrene ((C_8_H_8_)*n*) and polyvinylidene difluoride ((C_2_H_2_F_2_)*n*-)) polymers proved to be viable polymers when mixed in the correct proportion with Xylene (C_8_H_10_) and Dimethylformamide(C_3_H_7_NO), respectively. Although the latter has in its constitution oxygen, Mg_2_Si is not sensitive to O_2_ (only to moisture) and it is one of the most recommended solvents for effectively dissolving PVDF. Hence, using this polymer–solvent combination only requires assuring that the fraction of unreacted Mg and Si in the synthetized Mg_2_Si powder batch is inexistent or negligible. Also, PVDF should not be discarded because of its difficulty in finding compatible solvents without oxygen, since it has attractive properties for the Mg_2_Si pastes formulation. It is non-toxic, has good thermal stability up to 100 °C, melts at 170 °C, is resistant to chemicals, may exist in different crystalline forms depending on the preparation conditions, and most importantly, it has low water absorption characteristics [13]. Therefore, both polymeric solutions were mixed with the synthetized Mg_2_Si powders for obtaining *n*-type thermoelectric Mg_2_Si paste formulations. Prior to the PVDF paste formulation, three polystyrene paste formulations were derived and extruded by 3D printing, spray and electrospinning. Each paste formulation is described in Table 2, and the ability of producing 3D printed pieces, with a variety of shapes and high finishing quality, and fibers is illustrated in Fig. 5.

**Table 2:** Mg_2_Si paste formulations prepared with polystyrene (PS) solution for 3D printing and fiber production by spray and electrospinning

Paste ID	Paste application	PS solution: Mg_2_Si powder mass ratio	PS% wt. in xylene
1	3D printing	57:43	20
2	3D printing and spray (diluted)	60:40	20
3	Electrospinning	93:7	20

**Fig. 6:**
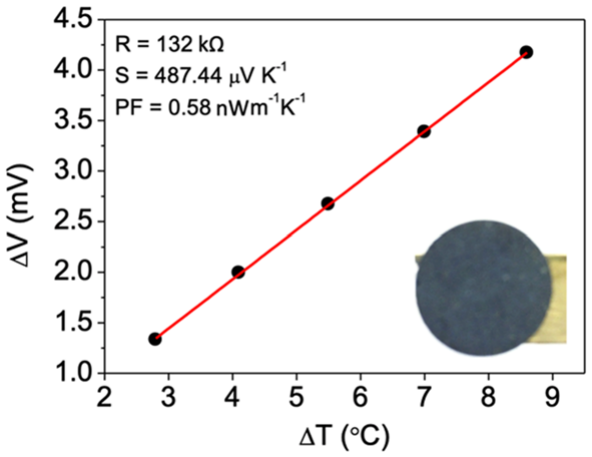
Thermo-voltage vs. temperature difference plot measured from an Mg_2_Si pellet. From the linear fit slope, the Seebeck coefficient was determined and enabled the calculus of the power factor, being both values indicated

For 3D printing, the paste was made of Mg_2_Si powders obtained from different milling times (5 h and 2 h). Figure 6a shows that the pieces produced with 5 h milled powder of lower grain size (formulation 1) are less smooth and have a worst finishing than those produced with higher grain size powder and with 3% less amount of Mg_2_Si powder (formulation 2). This latter formulation was next diluted for producing Mg_2_Si fibers by low N_2_ pressure spray with the Wuto gun and by electrospinning (formulation 3). The SEM images of the sprayed fibers are shown in Fig. 6b, c and evidence that these are aligned and incorporate Mg_2_Si grains. Similarly, the SEM images of electrospun fibers shown in Fig. 6c, d reveal their elongated beaded-like morphology, that includes Mg_2_Si aggregates. The Raman spectra measured on the printed pieces and sprayed fibers obtained with paste formulations 2 and 3, respectively, are also included in Fig. 6. The Raman peaks at ~ 251 cm^−1^ are assigned to Mg_2_Si phonon peaks identified in the literature as arising at 256–260 cm^−1^, being the shift probably due residual strain [14, 15].

The porosity of three printed small cylindrical pieces (made with formulation 2) was also determined by means of BET measurements, preceded by a standard 10 h thermal treatment. Two pieces, A and B, were heated at 120 °C and one at 300 °C, piece C. BET measurements on pieces A and B yielded a mean specific surface area of 4.11 ± 0.67 m^2^/g, a total pore specific volume of 0.0030 ± 0.0002 cm^3^/g, and a pore size of 6.00 ± 0.34 nm, while for piece C, these values proved to be significantly smaller: 14.9 ± 2.4 m^2^/g, 0.0194 ± 0.0013 cm^3^/g and 7.20 ± 0.41 nm, respectively. The reason is mainly attributed to the polymer evaporation that according to the literature is foreseen to occur at 210–249 °C, and at ~ 470 °C as determined by DSC/TGA measurements. Temperature at which polymer removal is expected to be completed. Such porosity after the polymer removal, led as expected, to a low thermal conductivity 0.226 ± 0.001 W/mK, that is significantly lower than the experimental (range from 7.8 to 4.0W/mK at 323 K and 623 K, respectively, [16]) and theoretical (~ 9.5 to 10.5 W/mK at 300 K [17]) values reported to Mg_2_Si, but led to insulating and mechanically fragile pieces. For that reason, a new paste formulation was devised. This was constituted by a solution of PVDF in DMF high boiling point solvent (153 °C). The aim was to prevent larger pores formation due to rapid solvent evaporation before and during the annealing for the polymer removal. Besides, the amount of polymeric solution in the paste was reduced. The mass ratio of PVDF solution to Mg_2_Si powder was optimized to 8/92 (formulation 4), which immediately led to the production of bulk Mg_2_Si thermoelectric pieces with electrical resistance and impressive thermoelectric properties. The polymeric solution is constituted by 6.6% wt of PVDF in DMF.

### Thermoelectric characterization

The thermoelectric characterization of Mg_2_Si powder and bulk pieces produced with the ‘PVDF in DMF’ formulation is next presented.

### Mg_2_Si pellet

Approximately, 283 mg of Mg_2_Si powder was 15 ton pressed to form a pellet with a diameter of ~ 12.84 mm and a thickness of ~ 1.55 mm. Measurements of the pellet electrical resistance and voltage under a temperature difference was plotted as shown in Fig. 6 for determining the Seebeck coefficient, where the former one corresponds to 132 kΩ and the later to 487 μV/K. This Seebeck coefficient value is in line with those reported in the literature for sintered Mg_2_Si, circa 500 μV/K [18], assuring that the Mg_2_Si powder herein synthetized for formulating the pastes is thermoelectric. The measured Seebeck coefficient value is slightly smaller, because of the formation of other minor phases, that are: unreacted Si and Mg, and SiO_2_ and MgO—as previously concluded, the powder is not pure Mg_2_Si. The curve of Fig. 6 not only enabled the calculus of the Seebeck coefficient, but also of the power factor, ~ 0.58 nW/mK.

### Mg_2_Si pieces made with PVDF polymeric solution

**Fig. 7:**
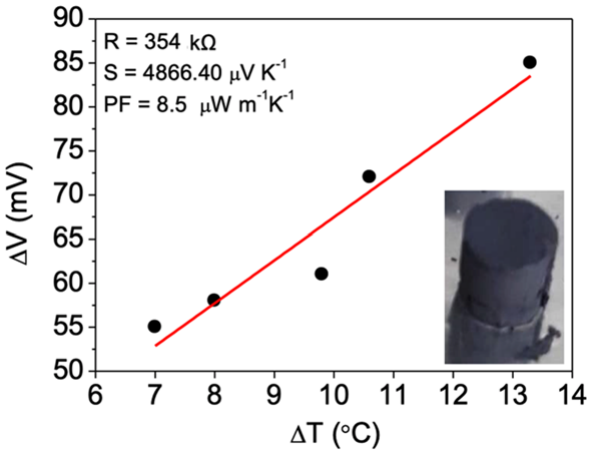
Thermo-voltage vs. temperature plot measured from a bulk Mg_2_Si piece made with PVDF in DMF solution

**Fig. 8:**
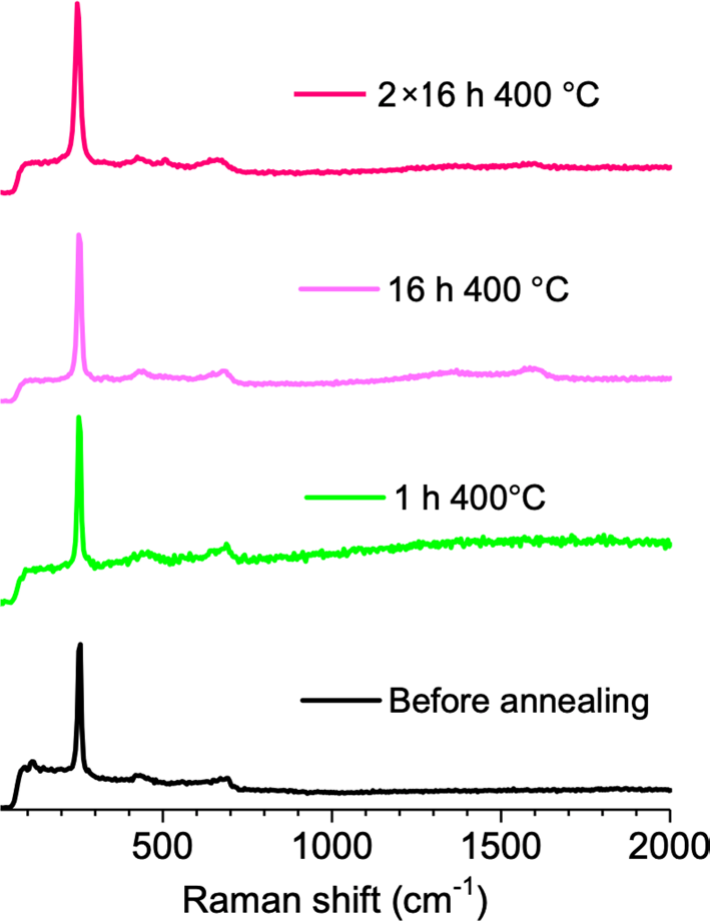
Raman spectra measured from replicas Mg_2_Si pieces made of 92% of Mg_2_Si and 8% of solution of PVDF in DMF before and after sintering performed at 400 °C of different durations: 1 h, 16 h and 32 h. Black and green curves correspond to one sample before and after sintering and pink curves correspond to another sample, first sintered by 16 h and next to another 16 h sintering (the 32 h were not followed)

Figure 7 shows the thermo-voltage measured as a function of the temperature difference applied to a bulk cylindrical piece made of paste constituted by 92% of Mg_2_Si and 8% of solution of PVDF in DMF prior sintering. This curve enabled the calculus of the Seebeck (~ 4866.40 μV/K) coefficient and of the power factor (~ 8.5 μW/mK) as illustrated in Fig. 7. The first is in the range of competitor materials such as BiTe and CoSb_3_ (~ 290–700 V/K) but contrary to CoSb_3_ (5 μW/mK), BiTe still presents a higher power factor (~ 0.25 mW/mK) than that of the Mg_2_Si bulk piece prior to the polymer removal. This is evidently contributing to the thermoelectric properties measured from such Mg_2_Si bulk piece. A few days later, the polymer inside the piece must have degraded, because it was no longer possible to measure the electrical resistance from this sample. Porosity initially filled by the polymer must have been partially undone due to the polymer degradation, which prevented the electrical resistance measurement. According to the literature, during useful life, polymers may be influenced by heat, oxygen, sunlight, mechanical stress, etc. Also solvents can be photo-oxidized, hydrolyzed or thermally decompose, and as a result degrade the polymer. Therefore, it is difficult to exactly determine what may have caused the PVDF polymer degradation and hence the lack of thermoelectric property stability before sintering. This piece and other new replicas were sintered through a pressureless sintering method that consisted in a long thermal annealing performed at 400 °C under inert atmosphere. However, this sintering method, which cannot be combined with mechanical pressure, proved to be ineffective, without leading to the consolidation of the bulk Mg_2_Si pieces produced with a mean grain size of 2–3 μm, even after two steps of 16 h. The porosity and strength properties were not sufficiently enhanced since after each sintering attempt, it was not possible to measure the electrical resistance. The sample composition was not significantly altered for all sintering attempts performed at 400 °C up to 32 h. The Raman spectra in Fig. 8 show that the dominant phase remains that of Mg_2_Si (247 cm^−1^ peak) which coexists with very small amounts of MgO (1348 and 1577 cm^−1^ peaks). Above 400 °C, Mg_2_Si is not thermally stable and other compounds start to form ~ MgO at 465 °C, ~ SiO_2_ at 710 °C and ~ Mg_2_SiO_4_ at ~ 1000 °C.

An alternative pressureless method that predictably may be used is the hot isostatic pressing sintering method, compatible with 3D shapes. This subjects a sample to both elevated temperature and isostatic gas pressure in a heated high pressure vessel filled with an inert gas for avoiding chemical reactions. This synthesis method may be an alternative that together with others may be worth to explore to overcome this sintering challenge and become the silicide’s extrusion viable in the production of bulk thermoelectric parts.

## Conclusions

Magnesium silicide powders, Mg_2_Si, for TE applications were successfully synthesized by combining ball milling and thermal annealing. Ball milling alone does not yield Mg_2_Si, as evidenced by XRD analysis, but it is needed to homogenize the particle size distribution and bring its overall dimensions to values suitable for their use in pastes compatible with techniques such as 3D printing, spray and electrospinning.

Although we have proven that Mg_2_Si may also be synthetized directly by thermal annealing only (without milling), this option requires higher processing temperature and revealed to be not compatible with some 3D printers operating with needles of inner diameter ≤ 1.19 mm.

The formulation of Mg_2_Si pastes with polystyrene and xylene proved to be viable for producing thermoelectric parts with varied shapes and fibers by means of 3D printing, low-pressure spray and electrospinning. Despite feasible, the PS-based paste 3D printing gives rise to very porous pieces, which hindered electrical and thermoelectrically characterization. As a result, the Mg_2_Si content in a new paste formulation was significantly increased and the polymeric solution changed to PVDF in DMF solution. This proved to be a viable formulation to generate bulk Mg_2_Si pieces with good thermoelectric properties (i.e. large Seebeck coefficient of 4866 μV/K and power factor of 8.5 μW/mK). However, the performance suffers from degradation over time, probably due to changes in the polymer properties. Also, the long pressureless sintering, performed at 400 °C due to the low thermal stability Mg_2_Si, has not been successful, which demonstrated that this sintering method does not allow consolidating the 3D pieces through pores reduction. Definitely, other alternatives will have to be explored to enable the silicides’s pastes formulation to be used in multiple extrusion techniques such as 3D printing and fiber making, which requires further optimization.
